# Comparison of New Kairomone-Based Lures for *Cydia pomonella* (Lepidoptera: Tortricidae) in Italy and USA

**DOI:** 10.3390/insects12010072

**Published:** 2021-01-15

**Authors:** Michele Preti, Alan L. Knight, Riccardo Favaro, Esteban Basoalto, Marco Tasin, Sergio Angeli

**Affiliations:** 1 Faculty of Science and Technology, Free University of Bozen-Bolzano, Piazza Università 5, 39100 Bolzano, Italy; michele.preti@natec.unibz.it (M.P.); riccardo.favaro@unibz.it (R.F.); 2 Instar Biologicals, Yakima, WA 98908, USA; uncfencer76@hotmail.com; 3 Facultad de Ciencias Agrarias, Instituto de Producción y Sanidad Vegetal, Universidad Austral de Chile, Valdivia 5110566, Chile; esteban.basoalto@uach.cl; 4 Department of Chemical Sciences, University of Padua, Via Marzolo 1, 35121 Padua, Italy; marco.tasin@unipd.it

**Keywords:** codling moth, sex pheromone, pear ester, acetic acid, dimethyl nonatriene, pyranoid linalool oxide, monitoring

## Abstract

**Simple Summary:**

Adult codling moth (*Cydia pomonella* L.) monitoring with lure-baited traps is a prerequisite to effectively manage this key pest in apple and pear crops without over-spraying insecticides. We evaluated new multi-component lures comprised of blends of sex pheromone and volatile organic compounds (pear ester, dimethyl nonatriene and linalool oxide) loaded into different substrates (septa and PVC lures). Acetic acid in a second membrane lure was used as a co-lure with all blends. Lure comparisons were performed during the period 2019/2020 in Italy and Washington State (USA) in orchards treated with or without sex pheromone dispensers for mating disruption. The highest total moth counts occurred with the sex pheromone/pear ester PVC lure in both countries. The new multi-component PVC lure without sex pheromone captured the greatest number of female moths only in the USA. This geographical disparity may limit the effectiveness of using a ‘female removal’ strategy to manage this pest without insecticides across major production areas.

**Abstract:**

Studies were conducted during the period 2019/2020 to evaluate the effectiveness of four lures for codling moth (*Cydia pomonella* L.) in pome fruits in Italy and the USA. Multi-component blends of sex pheromone ((*E*,*E*)-8,10-dodecadien-1-ol, PH), pear ester ((*E,Z*)-2,4-ethyl decadienoate, PE), (*E*)-4,8-dimethyl-1,3,7-nonatriene (DMNT), and pyranoid linalool oxide (6-ethenyl-2,2,6-trimethyloxan-3-ol, LOX) were loaded in either a halobutyl elastomer septum or a PVC matrix and always used in combination with acetic acid (AA) loaded in a closed membrane co-lure. Total moth capture was significantly greater with the PVC than the septum lure loaded with PH/PE + AA in both countries. Female capture in the USA study was significantly greater for 8 weeks in traps baited with the PE/DMNT/LOX blend + AA co-lure than with other lures and adding PH to this blend in a PVC lure significantly reduced female capture. In contrast, female capture in Italy did not differ among lures and counts were similar in both apple and pear crops treated with or without mating disruption. These results suggest that the effectiveness of ‘female removal’ strategies to manage codling moth may be geographically limited and further comparisons are needed in other production regions and in walnut.

## 1. Introduction

Codling moth, *Cydia pomonella* L. (Lepidoptera: Tortricidae), is a key world-wide pest of apple, *Malus domestica* L., pear, *Pyrus* spp., quince, *Cydonia oblonga* Mill., and walnut, *Juglans regia* L. [1,2]. The chemical ecology of the codling moth has been well investigated with several applications adopted by growers on a global scale for both monitoring and direct management [3,4,5]. The most important application has been the use of the female sex pheromone, (*E*,*E*)-8,10-dodecadien-1-ol (PH), in lures to monitor male moth population dynamics [6,7], and within various dispensers, aerosols, and sprayable formulations for mating disruption (MD) [8,9,10]. Monitoring codling moth with PH lures within MD-treated orchards is challenging because male sexual behaviors are affected and moth captures in traps can be imprecise and variable [11,12]. The improved monitoring of codling moth in MD-treated orchards has been achieved with the use of pear ester, (*E,Z*)-2,4-ethyl decadienoate (PE), in combination with PH [13]. The use of additive or synergistic compounds, such as acetic acid (AA) [14] and (*E*)-4,8-dimethyl-1,3,7-nonatriene (DMNT) [15], with PE or PH/PE has increased the capture of codling moth and allowed female population densities to be tracked [16,17,18,19]. Recently, Knight et al. [20] reported that the addition of pyranoid linalool oxide, 6-ethenyl-2,2,6-trimethyloxan-3-ol (LOX), to the blend of PE + DMNT + AA significantly outperformed other non-pheromone and pheromone blends in untreated and MD-treated orchards [21]. The addition of PH to the PE + DMNT + LOX + AA blend significantly increased total moth captures in MD-treated orchards. Both blends with or without PH were highly attractive to female codling moth, i.e., females comprised > 60% of the total captures [20,21]. The nearly 4-fold increase in female captures compared to other lures [22] can promote the further development and greater adoption of ‘female removal’ as a viable management practice for codling moth [23]. To date, the effectiveness of these new multi-component blends has not been reported outside the USA or in host crops other than apple.

Previous field studies with PE-based lures found that their relative effectiveness in trapping both sexes of the codling moth was variable, and a number of significant factors were identified, including seasonality, trap height and color [24,25,26,27,28,29]. Of particular concern were reports of low female captures in PE-baited traps in some geographical regions [30,31,32,33,34,35,36,37,38,39,40,41]. Gray halobutyl elastomer septa were used as the matrix for PH and PE lures to provide extended effectiveness compared to red rubber septa [11,18,19,25,42]. A new black PVC proprietary matrix developed for long-lasting mating disruption dispensers has also been developed for a range of insect lures [43].

Here, we report the first study of the PVC matrix used for codling moth lures loaded with PH, PE, DMNT, and LOX, and used in combination with an AA co-lure. Studies were conducted in both apple and pear orchards treated with or without MD in Italy and in an untreated apple orchard in the USA during the period 2019/2020.

## 2. Materials and Methods

### 2.1. Trapping Material and Lures

Standard orange delta-shaped traps with sticky liners were used in all studies (Pherocon^®^ VI, Trécé Inc., Adair, OK, USA). Two commercial lures were used in our studies, a grey halobutyl elastomer septum loaded with 3.5 mg PH and 3.9 mg PE (Pherocon^®^ CM-DA Combo), and a white plastic closed membrane lure loaded with 720 mg of AA (Pherocon^®^ AA). Three new black PVC lures were also tested. The first PVC lure was loaded by Trécé Inc. with the same rates of compounds (PH/PE) as the septum lure (Pherocon^®^ CM-DA Combo-P). Two experimental PVC lures were either loaded by Trécé Inc. with 3.9 mg PE, 10 mg of DMNT and 10 mg LOX or these same three compounds plus 3.5 mg PH.

### 2.2. Lure Comparison Field Trials

Lure comparisons were conducted during 2019 in one apple orchard not treated with MD in Washington State, USA, from 4 May to 22 July. The objective of this study was to determine the longevity of the lures over 4, 8, and 12 weeks of field exposure to reflect typical replacement schedules used with various alternative lures. In this lure longevity study, the lures were not replaced over the whole monitoring period (79 days) and codling moth captures were summarized on a monthly basis (ca. every four weeks) to assess the lures’ performance over different time periods. Studies were also conducted in four apple and three pear plots in the Emilia-Romagna region, Italy, during the period 2019/2020, to evaluate lures in both crops and in orchards with or without MD (thirteen trials). In Italy, pome fruit orchards were either unsprayed (one apple plot and two pear plots) or managed according to the organic farming practices plus MD (three apple plots and one pear plot). Isomate^®^-C TT (Shin-Etsu Chemical Co. Ltd., Chiyoda District, Japan) at 500 dispensers ha^−1^ (313 mg PH per dispenser) was applied in MD-treated apple plots, while the pear plot was treated with Cidetrak^®^ Meso (Trécé Inc.) at 80 dispensers ha^−1^ (1100 mg PH per dispenser). The purpose of the lures’ evaluation study realized in Italy was to gain information about the lures’ performance outside the USA and in different operative conditions. Trial duration varied from 3 to 12 weeks, covering the three codling moth flight periods (see Table S1 in Supplementary Material for details).

Experimental protocols were mostly standardized across the studies. Four blends were compared in every field trial: (1) the PH/PE blend loaded in a septum lure + AA membrane co-lure; (2) the PH/PE blend loaded in a PVC lure + AA membrane co-lure; (3) PE/DMNT/LOX blend loaded in a PVC lure + AA membrane co-lure; and (4) PH/PE/DMNT/LOX blend loaded in a PVC lure + AA membrane co-lure. All lures were placed directly on the center area of the sticky liner placed inside the bottom of the trap. Traps with no lures were included as an untreated blank in each trial. Traps were placed within each orchard plot with a completely randomized experimental design and were spaced at least 25 m apart and from the orchard’s perimeter. Each trap was attached to a bamboo pole to facilitate their placement at a height of 2.5–3.0 m in the canopy. Traps were checked, captures were counted and sexed, and liners were replaced weekly. Traps were not rotated in position during each trial. The number of replicates varied across studies: 8 traps per each of the 5 treatments in the USA lure longevity trial and 5 traps per each of the 5 treatments in each of the 13 Italian lure comparison trials.

### 2.3. Statistical Analyses

Statistical analyses of moth counts (females and total) were performed with R software version 4.0.3 (R Core Team 2020) [44], including the packages lme4 [45] and multcomp [46]. Figures were created using the package ggplot2 [47]. For the USA data, the effect of lure type on the codling moth captures was analyzed by using linear models (lm), after data transformation with a square root (x) to fit a normal distribution. The Italian data were analyzed by using generalized linear models (glm) or generalized linear mixed-effects models (glmer) with Poisson distribution, thus accounting for the variability within each trial. The models were tested for overdispersion (AER package). A negative binomial distribution (glm.nb function from the MASS package) was used in case of overdispersed models. Akaike’s information criteria (AIC) and residuals were used to select the fitted models. Data from traps without lures were always excluded from the statistical analysis because these traps failed to capture moths during any trial. The Italian data were analyzed both together and separately for each crop and the use of MD. For the comprehensive analyses, a glmer with a Poisson distribution tested the effects of several factors on moth captures. Codling moth flight, trial duration, MD occurrence, grower management program (either unsprayed or organic), crop (either apple or pear) and year were considered as predictors together with the lure type, while the trial number was set as a random effect. The significance of the random effect was tested against a dummy model without a random effect. A multiple comparison post hoc test was performed on the fitted models (glht function from multcomp package) for both USA and Italian data and Tukey’s test (*p* < 0.05) was adopted to discriminate differences.

## 3. Results

### 3.1. Lures Longevity Study (USA)

**Figure 1:**
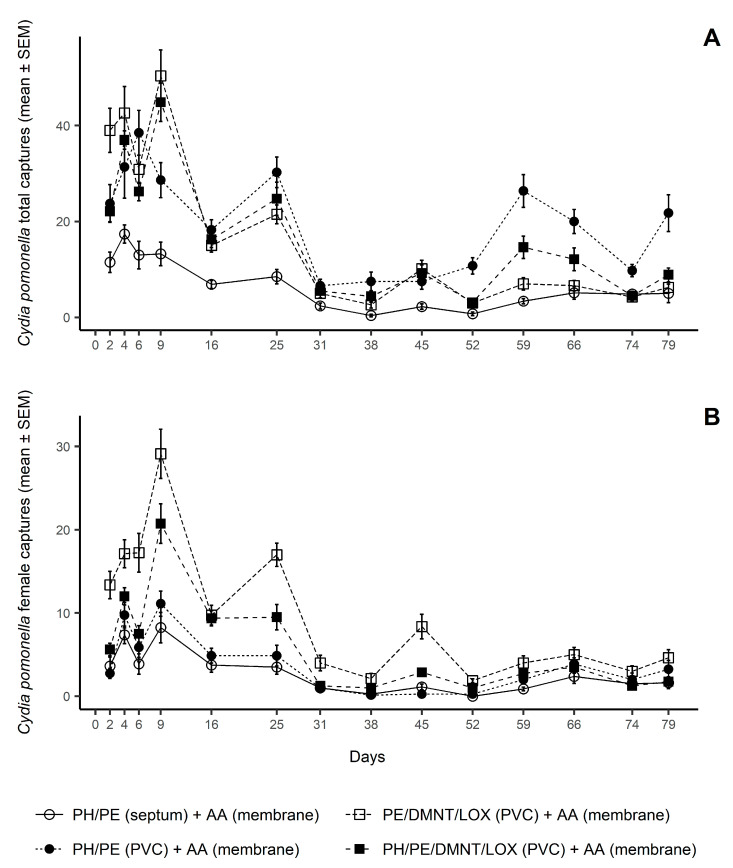
Mean (±SEM) codling moth (*Cydia pomonella* L.) captures in the weekly trap checks recorded in the USA from 4 May to 22 July 2019. (**A**) = total captures; (**B**) = female captures. This lure longevity study tested four blend combinations of (*E*,*E*)-8,10-dodecadien-1-ol (sex pheromone, PH), (*E,Z*)-2,4-ethyl decadienoate (pear ester, PE), acetic acid (AA), (*E*)-4,8-dimethyl-1,3,7-nonatriene (DMNT), and linalool oxide pyranoid (LOX), over a 12-week monitoring period (N = 8).

Traps captured large numbers of codling moths during the lure longevity study performed in the USA in 2019 (Table 1 and Figure 1). All three PVC lures captured significantly more moths than the PH/PE septum in the first two time periods. Interestingly, the PH/PE PVC lure captured significantly more males (between 3.3-fold and 9.3-fold) than the PH/PE septum lure during all the experiment. The two PVC lures with DMNT and LOX captured significantly more females than either PH/PE lures in the first two time periods. The addition of PH in the multi-component PVC lure significantly reduced the capture of females in both time periods. The relative performances of the lures changed in the third period. After 8 weeks of field exposure, the PH/PE PVC lure still captured significantly more total moths than the other three lures, but the multi-component PVC lure without PH only captured significantly more females than the PH/PE septa lure. Over the entire 79 days of the experiment, the three PVC lures captured significantly more moths than the septum lure, the two PVC lures with DMNT and LOX captured significantly more females than the two PH/PE lures, and the addition of PH in the multi-component PVC lure reduced female captures (Table 1). The multi-component PVC lure without PH provided the highest female counts, with a final female proportion (mean ± SEM) of 0.56 ± 0.02, significantly greater than the female proportion of 0.35 ± 0.02 provided by the same blend plus PH (df = 3, 28; F = 30.33; *p* < 0.001).

### 3.2. Lures Evaluation in Different Field Conditions (Italy)

Moth counts in the Italian unsprayed apple orchard were at least 2-fold higher than the ones recorded in the apple orchards treated with MD, while moth counts in the pear orchard treated with MD doubled the counts recorded in the unsprayed pear orchards (Table 2).

The PH/PE PVC lure captured significantly more total moths than other lures only in the apple trial without. The multi-component PVC lure with PH captured significantly fewer moths than the PH/PE lures in no-MD pear trial. The multi-component PVC lure captured significantly more females than the PH/PE septum lure in the no-MD apple trial and both the PH/PE PVC and multi-component with PH added PVC lures in the pear-MD trials. The PH/PE PVC lure captured significantly more females than the septum lure in the apple no-MD trial.

Comprehensive data analysis of Italian trials (df = 219) showed no significant effect of codling moth flight (z value = 0.014 and *p* = 0.989; z value = 0.853 and *p* = 0.394 for the second and third flight, respectively), trial duration (z value = −1.719 and *p* = 0.086), MD occurrence (z value = −0.936 and *p* = 0.349), year (z value = 1.372 and *p* = 0.170), and crop (z value = 1.426 and *p* = 0.154) on the total codling moth captures. The same result was obtained for female and male captures. In the comprehensive analysis, the PH/PE PVC lure captured significantly more total moths than either of the PVC lures including DMNT/LOX. This significant difference was due to the male captures, which used the PH/PE PVC lure and were 1.5-fold higher than using the same PH/PE blend in the septum lure, both in combination with an AA co-lure. On the contrary, female captures were similar for all lures (Table 2). The multi-component PVC lure without PH provided the highest female counts, with a female proportion (mean ± SEM) of 0.38 ± 0.04, not different from the female proportion of 0.33 ± 0.04 provided by the same blend plus PH (df = 3, 201; F = 10.42; *p* = 0.997).

## 4. Discussion

Studies conducted in Italy and the USA with the new multi-component PVC lures provided contrasting results between countries and with the earlier reports of blend activities [20,21]. The original discovery used separate lures for each of the five compounds compared with the new PVC lure in which three or four compounds were loaded together; and both studies used the same AA membrane co-lure. Here, we found that adding PH to the multi-component PVC lure significantly reduced female codling moth captures in apple in the USA; but a similar effect in Italy was only seen in pear treated with MD dispensers. Previously, no difference in male or female captures were found if PE and PH were loaded into separate lures or combined in one septum [12,31]. Unfortunately, several studies evaluating the PE–PH combination in other countries did not make this exact comparison [33,36,38]. Our data here suggest that loading PH with the three plant volatiles in the PVC lure would diminish the potential of using ‘female removal’ to suppress codling moth populations, unless PH is released from a separate lure. However, further studies comparing these blends with PH released either by the same lure matrix including the three other compounds or by a close lure should confirm this hypothesis.

Our current study found that the new PVC matrix outperformed the septum lure with a PH/PE loading plus the use of the AA co-lure in both countries. However, only in Italy did the four lures capture similar numbers of female codling moths. Following the development of PE as an attractant for codling moths, at least one study in Italy suggested that PE might not be so effective for female moths [48]. However, more extensive electrophysiological, oviposition, and trapping studies in Italy demonstrated that the female codling moth did respond to PE similarly to populations in the USA [30,35,49]. Our new results suggest that different geographical populations of codling moth may have responses to host plant or microbial volatiles as variable as moth species have shown with sex pheromones [50,51].

PE is a major volatile component from ripe pears, and an initial hypothesis was made that it would be less effective in pear orchards due to volatile masking [24]. However, a series of studies demonstrated that PE was an effective lure for codling moth in many cultivars of pear [31,37,38,52,53]. PE was only found to be less effective in pear in one study under a specific set of conditions: late in the season in ‘Bartlett’ pear orchards characterized by high moth counts in traps and the presence of fruit injury [53]. In Italy, both for total moth captures and the proportion of females captured we found no apparent difference in the performance of the new multi-component lures between apple and pear.

The emission rate and blend ratios likely change continuously over time with any multi-component lure [54]. The USA study showed that the four-component PVC lure appeared to have diminished attraction for codling moths after 6 weeks when compared with the standard PE–PH lure. Previous studies have shown that DMNT in a grey halobutyl septum was an effective attractant for only 3–4 weeks [18], likely due to a rapid loss in the residual content of field-aged lures, i.e., 50% drop in 7 days [16]. However, both PE/PH and AA lures have been shown to be long lasting [19,29]. The emission rate of LOX from lures has not been reported. Eliminating DMNT from the four-component blend can reduce the lure’s attractiveness 3-fold [20]. Thus, it appears that the four-component lure gradually loses its effectiveness as the contribution of DMNT in the blend is reduced. Further studies are needed to assess and confirm this hypothesis, evaluating the emission rates of the various components loaded into the PVC matrix over time.

The failure of the four- and five-component lures in Italy to provide superior performance to the standard PH/PE + AA lure suggests that additional host plant volatile blends in combination with AA should be investigated. Extensive work in the USA has identified several four-component blends with PE, DMNT, and AA plus one of several terpenes from apple, pear, and walnuts, i.e., β-myrcene, β-pinene, α-farnesene and β-farnesene [55]. Similar studies should be conducted in Italy and other production areas to validate the effectiveness of these new blends prior to developing ‘female removal’ tactics.

## 5. Conclusions

Reliable surveillance of codling moth flight provides useful information for the timely management of pest population and limit crop losses. However, only monitoring male moths requires a robust correlation with key life history events, i.e., female egg laying and larval eclosion [56]. Thus, monitoring female populations should be a more accurate approach to predict key events [57,58]. The recent availability of new kairomone-based blends such as the four-component lure with enhanced formulations (PVC dispensers instead of septa) will likely open new approaches to monitor and manage this important pest. However, inherent behavioral differences among geographical populations of codling moth will need to be considered in this development.

## Figures and Tables

**Table 1:** Mean (±SEM) cumulative captures of codling moths in traps baited with four different combinations of (*E*,*E*)-8,10-dodecadien-1-ol (sex pheromone, PH), (*E*,*Z*)-2,4-ethyl decadienoate (pear ester, PE), (*E*)-4,8-dimethyl-1,3,7-nonatriene (DMNT), and linalool oxide (LOX) loaded into either an elastomer septum or a PVC matrix, and used in combination with a closed membrane lure loaded with acetic acid (AA), (N = 8) over three consecutive time periods from 4 May to 22 July 2019, USA.

Start–End of Trapping Period (d)	Lure Loading and Matrix ^a^	Mean (±SEM) Moth Capture per Trap		
		Total	Females	Males
0–31	PH/PE (septum) + AA	72.9 ± 6.3 a	31.4 ± 3.5 a	41.5 ± 3.6 a
	PH/PE (PVC) + AA	177.4 ± 14.7 b	40.3 ± 5.1 a	137.1 ± 15.7 b
	PE/DMNT/LOX (PVC) + AA	204.4 ± 16.4 b	107.6 ± 7.8 c	96.8 ± 9.9 b
	PH/PE/DMNT/LOX (PVC) + AA	176.8 ± 11.6 b	66.0 ± 3.7 b	110.8 ± 9.9 b
	ANOVA: df = 3, 28	*F* = 35.95, *p* < 0.001	*F* = 33.92, *p* < 0.001	*F* = 26.61, *p* < 0.001
32–59	PH/PE (septum) + AA	9.2 ± 1.5 a	3.3 ± 0.8 a	5.9 ± 1.2 a
	PH/PE (PVC) + AA	58.8 ± 7.1 c	3.6 ± 0.8 a	55.1 ± 6.9 c
	PE/DMNT/LOX (PVC) + AA	27.8 ± 3.1 b	20.4 ± 2.7 c	7.4 ± 0.8 a
	PH/PE/DMNT/LOX (PVC) + AA	36.9 ± 5.2 bc	8.9 ± 0.7 b	28.0 ± 5.3 b
	ANOVA: df = 3, 28	*F* = 41.01, *p* < 0.001	*F* = 26.12, *p* < 0.001	*F* = 54.74, *p* < 0.001
60–79	PH/PE (septum) + AA	15.0 ± 3.4 a	5.5 ± 2.0 a	9.5 ± 3.2 a
	PH/PE (PVC) + AA	51.5 ± 5.2 c	9.1 ± 2.1 ab	42.5 ± 5.1 c
	PE/DMNT/LOX (PVC) + AA	17.1 ± 1.9 ab	12.6 ± 1.6 b	4.4 ± 0.7 a
	PH/PE/DMNT/LOX (PVC) + AA	25.5 ± 3.5 b	6.5 ± 1.2 ab	18.8 ± 3.0 b
	ANOVA: df = 3, 28	*F* = 14.75, *p* < 0.001	*F* = 3.87, *p* = 0.020	*F* = 23.44, *p* < 0.001
0–79	PH/PE (septum) + AA	94.6 ± 9.5 a	39.1 ± 4.4 a	55.5 ± 7.1 a
	PH/PE (PVC) + AA	281.0 ± 19.9 b	52.0 ± 5.2 a	229.1 ± 22.6 c
	PE/DMNT/LOX (PVC) + AA	244.3 ± 17.8 b	136.6 ± 9.2 c	107.5 ± 9.9 b
	PH/PE/DMNT/LOX (PVC) + AA	233.6 ± 16.3 b	80.1 ± 4.8 b	153.3 ± 14.4 bc
	ANOVA: df = 3, 28	*F* = 42.40, *p* < 0.001	*F* = 39.15, *p* < 0.001	*F* = 34.60, *p* < 0.001

Column means within each period followed by different letters are significantly different, *p* < 0.05 (Tukey’s test). ^a^ The acetic acid loaded in a membrane co-lure was placed close to the other lure in all traps.

**Table 2:** Cumulative mean (±SEM) capture of codling moths in traps baited with different combinations of (*E*,*E*)-8,10-dodecadien-1-ol (sex pheromone, PH), (*E,Z*)-2,4-ethyl decadienoate (pear ester, PE), (*E*)-4,8-dimethyl-1,3,7-nonatriene (DMNT), linalool oxide (LOX) loaded into either an elastomer septum or a PVC matrix, and used in combination with a closed membrane lure loaded with acetic acid (AA), (N = 5). Thirteen lure comparison trials were conducted in apple and pear orchards either unsprayed or organically managed with mating disruption (MD) in Italy during the period 2019/2020.

Crop (Number of Trials)	Grower Management	Lure Content and Matrix ^a^	Mean (±SEM) Moth Capture per Trap		
			Total	Females	Males
Apple (1)	Unsprayed, no MD	PH/PE (septum) + AA	15.4 ± 5.4 a	3.0 ± 1.1 a	12.4 ± 4.6 ab
		PH/PE (PVC) + AA	38.4 ± 6.6 b	9.0 ± 1.0 b	29.4 ± 6.1 b
		PE/DMNT/LOX (PVC) + AA	12.0 ± 2.4 a	8.2 ± 1.8 b	3.8 ± 0.8 a
		PH/PE/DMNT/LOX (PVC) + AA	14.0 ± 7.0 a	6.6 ± 3.1 ab	7.4 ± 4.0 a
			Negative Binomial X^2^ = 13.83, *p* = 0.003	Poisson X^2^ = 18.03, *p* < 0.001	Negative Binomial X^2^ = 23.79, *p* < 0.001
Apple (6)	Organic, MD	PH/PE (septum) + AA	6.4 ± 1.3	1.6 ± 0.5	4.9 ± 1.0
		PH/PE (PVC) + AA	5.9 ± 1.4	0.5 ± 0.2	5.4 ± 1.4
		PE/DMNT/LOX (PVC) + AA	6.0 ± 1.1	1.9 ± 0.4	4.0 ± 0.8
		PH/PE/DMNT/LOX (PVC) + AA	6.1 ± 1.3	2.1 ± 0.5	4.1 ± 1.0
			Negative Binomial X^2^ = 2.61, *p* = 0.455	Negative Binomial X^2^ = 6.69, *p* = 0.083	Negative Binomial X^2^ = 7.76, *p* = 0.051
Pear (4)	Unsprayed, no MD	PH/PE (septum) + AA	9.9 ± 2.1 b	1.9 ± 0.5	7.9 ± 1.8 b
		PH/PE (PVC) + AA	11.5 ± 4.3 b	2.5 ± 1.2	9.0 ± 3.4 b
		PE/DMNT/LOX (PVC) + AA	7.6 ± 1.7 ab	1.9 ± 0.5	5.7 ± 1.6 ab
		PH/PE/DMNT/LOX (PVC) + AA	4.2 ± 1.1 a	1.3 ± 0.4	2.9 ± 0.8 a
			Negative Binomial X^2^ = 15.75, *p* = 0.001	Negative Binomial X^2^ = 2.32, *p* = 0.509	Negative Binomial X^2^ = 16.80, *p* < 0.001
Pear (2)	Organic, MD	PH/PE (septum) + AA	23.9 ± 5.8 bc	2.6 ± 0.4 ab	21.3 ± 5.5 b
		PH/PE (PVC) + AA	28.1 ± 3.7 c	1.2 ± 0.4 a	26.9 ± 3.6 b
		PE/DMNT/LOX (PVC) + AA	9.2 ± 1.6 a	3.7 ± 0.9 b	5.6 ± 1.1 a
		PH/PE/DMNT/LOX (PVC) + AA	11.4 ± 2.5 ab	1.6 ± 0.4 a	9.7 ± 2.2 a
			Negative Binomial X^2^ = 19.71, *p* < 0.001	Poisson X^2^ = 15.14, *p* = 0.002	Negative Binomial X^2^ = 31.79, *p* < 0.001
Apple and pear (13)	Comprehensive analysis	PH/PE (septum) + AA	10.9 ± 1.5 bc	2.0 ± 0.3	9.0 ± 1.3 b
		PH/PE (PVC) + AA	15.4 ± 2.3 c	2.0 ± 0.5	13.3 ± 2.0 c
		PE/DMNT/LOX (PVC) + AA	7.5 ± 0.8 ab	2.7 ± 0.4	4.8 ± 0.6 a
		PH/PE/DMNT/LOX (PVC) + AA	7.0 ± 1.0 a	2.1 ± 0.4	4.9 ± 0.7 a
			Negative Binomial X^2^ = 26.71, *p* < 0.001	Negative Binomial X^2^ = 4.87, *p* = 0.182	Negative Binomial X^2^ = 46.74, *p* < 0.001

Column means within each crop-management category followed by different letters are significantly different, *p* < 0.05 (Tukey’s test). X^2^ = chi-square value for the factor lure, *p* = probability *p*(X^2^) for differences among lures. ^a^ The acetic acid loaded in a membrane co-lure was placed close to the other lure in all traps.

## Data Availability

Data available on request.

## Supplementary Materials

The following are available online at https://www.mdpi.com/2075-4450/12/1/72/s1, Table S1: Summary of 14 lure trials for codling moths (*Cydia pomonella* L.) conducted in unsprayed and organic apple and pear orchards treated with or without mating disruption (MD) in Washington State, USA, and in the Emilia-Romagna Region, Italy, during the period 2019/2020.
