# Supplementary material for: Comparison of New Kairomone-Based Lures for Cydia pomonella (Lepidoptera: Tortricidae) in Italy and USA
Source: Insects. 2021 Jan 15;12(1):72. doi: 10.3390/insects12010072 (PMC7830130; doi:10.3390/insects12010072)
Supplement: Supplementary file 1 [file insects-12-00072-s001.pdf]

insects MDPI

Supplementary material

Table S1. Summary of 14 lure trials for codling moths (*Cydia pomonella* L.) conducted in unsprayed and organic apple and pear orchards treated with or without mating disruption (MD) in Washington State, USA, and in the Emilia-Romagna Region, Italy, during the period 2019/2020.

Orchard (Region, State) Location Coordinates	Crop, Cultivar, Surface	Grower Management	Trial Periods
Wapato (WA, USA) 46°24'33.08"N, 120°29'0.55"W	Apple, cv Delicious, 4 ha	Unsprayed (no-MD)	4 May–22 July 2019
San Bartolomeo in Bosco (FE, Italy) 44°42'53.69"N, 11°35'56.70"E	Apple, cv Fuji, 1.5 ha	Unsprayed (no-MD)	7 July–1 September 2020
San Cesario sul Panaro (MO, Italy) 44°34'2.39"N, 11°0'46.83"E	Pear, cv Abate Fetel and Williams, 0.5 ha	Unsprayed (no-MD)	17 July–16 August 2019<br>27 April–11 June 2020<br>12 June–8 August 2020
Baricella (BO, Italy) 44°38'54.28"N, 11°32'41.17"E	Pear, cv Abate Fetel and Decana, 0.4 ha	Unsprayed (no-MD)	11 July–16 August 2019
Villa Prati di Bagnacavallo (RA, Italy) 44°27'26.23"N, 12°1'55.47"E	Apple, cv Primiera, 3.5 ha	Organic (MD)	28 June–6 August 2019<br>6 August–16 September 2019
Villa Prati di Bagnacavallo (RA, Italy) 44°27'33.65"N, 12°1'43.48"E	Apple, cv Gold Rush, 3.0 ha	Organic (MD)	12 July–2 August 2019<br>2 August–16 September 2019
Villa Prati di Bagnacavallo (RA, Italy) 44°27'25.87"N, 12°1'57.71"E	Apple, cv Gaia, 1.5 ha	Organic (MD)	8 April–3 June 2020<br>10 June–30 July 2020
Renazzo (FE, Italy) 44°46'12.93"N, 11°18'8.00"E	Pear, cv Abate Fetel, 1.2 ha	Organic (MD)	10 April–5 June 2020<br>5 June–5 August 2020

Mating disruption (MD) was performed by using dispensers loaded with (*E,E*)-8,10-dodecadien-1-ol (sex pheromone).

Author Contributions: Conceptualization and methodology, M.P. and A.K.; investigation, M.P. (Italy), A.K., M.P. and E.B. (USA); data curation and statistical analysis, M.P. and R.F.; writing—original draft preparation, M.P.; writing—review and editing, M.P., R.F., E.B., M.T., A.K. and S.A.; supervision, S.A. All authors have read and agreed to the published version of the manuscript.

Funding: This research received no external funding.

Acknowledgments: The authors thank Trécé Inc. for supplying the lures and trapping materials.
